# Molecular basis of linezolid resistance in *Finegoldia* spp. from orthopedic infections

**DOI:** 10.1128/aac.01702-25

**Published:** 2026-02-26

**Authors:** Vincent Jean-Pierre, Mikeldi Moulieras, Alix Pantel, Fabien Aujoulat, Jean-Philippe Lavigne, Agathe Boudet, Hélène Marchandin

**Affiliations:** 1 HydroSciences Montpellier, Université Montpellier, CNRS, IRD, Service de Microbiologie et Hygiène Hospitalière, CHU de Nîmes 36672, Montpellier, France; 2 Service de Microbiologie et Hygiène Hospitalière, Université Montpellier, CHU Nîmes 36672, Nîmes, France; 3 VBIC, INSERM U1047, Université Montpellier, Service de Microbiologie et Hygiène Hospitalière, Plateforme MICRO&BIO, CHU Nîmes 36672, Nîmes, France; 4 HydroSciences Montpellier, Université Montpellier, CNRS, IRD, Nîmes, France; University of Fribourg, Fribourg, Switzerland

**Keywords:** *Finegoldia *sp., anaerobes, linezolid, resistance, *cfr*(C) gene, integrative and conjugative elements (ICE), bone infection

## Abstract

*Finegoldia* spp. are Gram-positive anaerobic cocci increasingly recognized as opportunistic pathogens, particularly in bone infections. Linezolid (LZD), an oxazolidinone with activity against anaerobes, is frequently used in orthopedic infections but is subject to various resistance mechanisms. Here, we characterized the molecular basis of LZD resistance in *Finegoldia* sp. isolates from three separate cases of bone infection. Antimicrobial susceptibility testing (AST) was performed according to Antibiotic Susceptibility Committee of the French Society of Microbiology (CA-SFM) recommendations. Whole-genome sequences were analyzed to challenge mass spectrometry identification (ANIb, *is*DDH), determine isolate relatedness (SNP analysis), and characterize the genetic support of resistance (ABRicate, AMRFinderPlus). LZD-resistant strains were isolated following LZD treatment in all three patients. In one case, low SNP divergence between susceptible and resistant isolates supported *in vivo* emergence of resistance. Genomic analyses suggested that the strains probably belong to undescribed *Finegoldia* species. LZD resistance was mediated by the *cfr*(C) gene encoding a 23S rRNA methyltransferase, and in two isolates with high-level resistance, was associated with a G71D mutation in the ribosomal protein L4-encoding gene. The *cfr*(C) gene was located on two distinct integrative and conjugative elements closely related to ones previously described in *Clostridioides difficile* and *Faecalibacterium taiwanense*. In anaerobes, LZD resistance remains rarely reported in the literature, except in non-*fragilis Bacteroides* species. However, LZD resistance in the *Finegoldia* genus highlighted in this study supports the need for systematic AST of *Finegoldia* sp. isolates, particularly in polymicrobial infections, when LZD is considered for the treatment. If LZD is ineffective, tedizolid testing may be suggested as a potential therapeutic alternative.

## INTRODUCTION

The *Finegoldia* genus was created in 1999 and comprises strictly Gram-positive anaerobic cocci (GPAC). Until 2024, it included a single species, validly named *Finegoldia magna*. In 2024, *Finegoldia dalianensis* was described by Li et al. following its isolation from a purulent discharge from a human skin abscess. Genomic analyses conducted in that study revealed significant heterogeneity within the genus, suggesting the probable existence of hitherto uncharacterized *Finegoldia* species (1).

*F. magna* is a commensal species of human microbiota that may also act as an opportunistic pathogen in humans (2). It is consistently the GPAC most commonly isolated from human clinical samples. Although usually recovered in polymicrobial cultures, *F. magna* is also the most common GPAC isolated as the sole pathogen in various infectious diseases, confirming its pathogenic power in humans (2). The main problems caused by *F. magna* are soft tissue and bone infections, including orthopedic implant-associated infections, a pattern that distinguishes it from other GPAC species (3–5). Genomic and phenotypic studies have revealed that *F. magna* possesses an arsenal of virulence factors acting at different stages of infection. These include pili for adhesion, biofilm formation for persistence, collagenase for tissue invasion, and pro-inflammatory properties that promote neutrophil activation and immune evasion (2, 6–9).

*F. magna* is usually regarded as a species susceptible to antibiotics with anti-anaerobic activity, except for clindamycin and moxifloxacin (3, 10, 11). Among the antibiotics active against *F. magna*, linezolid (LZD) is the first representative of the oxazolidinone family, approved for use in France since 2000–2001 for the treatment of community-acquired and healthcare-associated pneumonia when Gram-positive pathogens are documented or suspected, as well as for complicated skin and soft tissue infections. LZD is a bacteriostatic antibiotic that inhibits protein synthesis by targeting the bacterial ribosome (12). LZD lacks activity against Gram-negative aerobes but is active against anaerobes, both Gram-positive and -negative species, making it a valuable therapeutic option in the management of the aforementioned infections in which anaerobes might be involved. LZD is also frequently used off-label in bone and joint infections caused by Gram-positive aerobic cocci due to its broad activity spectrum and oral bioavailability despite concerns about side effects during prolonged treatments. Its activity against anaerobes is also particularly relevant in polymicrobial infections, such as diabetic foot osteomyelitis.

Resistance to LZD remains rare in the literature (3, 13–16), except in *Bacteroides* spp. (17). Mechanisms supporting this resistance, mostly studied in aerobes, encompass mutations in the 23S ribosomal RNA genes (mostly a G2576T substitution), mutations in ribosome-associated proteins (L3, L4), acquisition of 23S rRNA methyltransferases of Cfr type (for chloramphenicol-florfenicol resistance) causing methylation of rRNA-binding sites, and ribosomal protection through drug dissociation from the ribosome by ATP-binding cassette (ABC-F) proteins, such as OptrA and PoxtA, located on plasmids or chromosomes (15). Depending on the resistance mechanism and the combination of diverse resistance determinants, low-to-high-level resistance (minimal inhibitory concentrations [MICs] of LZD ranging from 8 to >256 mg/L) has been reported in the literature, together with the description of either isolated LZD resistance or, more frequently, multidrug-resistant phenotypes, such as PhLOPS_A_ (resistance to phenicols, lincosamides, oxazolidinones, pleuromutilins, and streptogramin A) and cross-resistance (or not) to tedizolid (18).

These observations, together with the marked increase in LZD use noted in France with a ninefold increase in commercial use between 2020 and now (19), support the need for surveillance on the emergence of LZD resistance. Here, we studied the molecular basis of LZD resistance in three cases of human bone infection involving *Finegoldia* sp. because it has not been explored before in the rare LZD-resistant GPAC strains reported in the literature.

## MATERIALS AND METHODS

### Patients and bacterial strains

Three patients (P1, P2, and P3) hospitalized for bone infections at Nîmes University Hospital (Southern France) between May 2021 and January 2025 were included in this study. Bone samples were processed according to national recommendations (20). Anaerobic cultures were performed on *Brucella* blood agar (BBA) plates supplemented with hemin and vitamin K1 (bioMérieux, Marcy l’Etoile, France) and incubated at 37°C under anaerobiosis using the Anaerobe Container System, GasPak EZ (Becton, Dickinson and Company, Sparks, USA) for 14 days. Five bacterial strains isolated from these three patients and stored frozen at −80°C were available for analysis. These included four *Finegoldia* sp. strains and one *Staphylococcus epidermidis* strain designated as follows: (i) P1-F-LS and P1-F-LR, for LZD-susceptible and LZD-resistant *Finegoldia* sp. strains, respectively, isolated sequentially in Patient P1; (ii) P2-F-LR for LZD-resistant *Finegoldia* sp. strain isolated in Patient P2; and (iii) P3-F-LR and P3-SE-LR for LZD-resistant *Finegoldia* sp. and *S. epidermidis* strains co-isolated in the same sample from Patient P3. Isolates were identified by matrix-assisted laser desorption/ionization time-of-flight mass spectrometry (MALDI-ToF MS) (Bruker Daltonics, Bremen, Germany) (MBT Compass HT IVD reference library, version 5.2.320, reference 1877017). Clinical and therapeutic data were retrospectively extracted from patient medical records, and microbiological data were retrieved from the bacteriology laboratory.

### Antimicrobial susceptibility testing

Antimicrobial susceptibility testing (AST) was performed according to the 2025 Antibiogram Committee recommendations of the French Society for Microbiology (CA-SFM) (21). For *Finegoldia* sp., E-test strips (bioMérieux) were used on BBA plates (bioMérieux), except for piperacillin-tazobactam and chloramphenicol, which were tested using disk diffusion (Bio-Rad, Marnes-la-Coquette, France). Reading was performed after 20 ± 4 h of incubation (44 ± 4 h for the search for inducible resistance to clindamycin and metronidazole) at 35 ± 2°C in anaerobic conditions (21). In addition, a D-test (erythromycin disk placed in proximity to the clindamycin disk) was used to detect inducible clindamycin resistance and specify the resistance phenotype to the macrolides-lincosamides-streptogramin B (MLSb) class. For P3-SE-LR, AST was performed by broth microdilution using the VITEK 2 AST-P668 card (bioMérieux). LZD resistance was confirmed using E-test strips (bioMérieux) on Mueller-Hinton agar (Bio-Rad) under aerobic conditions. For *Finegoldia* sp. and *S. epidermidis*, reading of LZD and tedizolid susceptibility assays was performed after 20 ± 4 and 44 ± 4 h of incubation in transmitted light (with the plate facing the light) (21). Resistance to LZD was defined as a MIC > 4  mg/L (21).

### Whole-genome sequencing and analysis

*Finegoldia* sp. isolates were cultured anaerobically at 37°C for 48 h on BBA plates (Becton Dickinson), whereas P3-SE-LR was grown aerobically at 37°C for 24 h on Columbia sheep blood agar plate (5%) (bioMérieux). Genomic DNA was extracted using the DNeasy UltraClean Microbial Kit (Qiagen, Aarhus, Denmark) according to the manufacturer’s instructions. Whole-genome sequencing libraries were prepared from 250 ng of extracted DNA using the Illumina DNA Prep Kit Library Paired-End Protocol (Illumina, San Diego, USA). A 250 bp paired-end sequencing run was performed on a MiSeq platform (Illumina) using MiSeq Reagent kit v2. Data quality was controlled directly on Miseq output reads using a QC platform (v0.23.2). Reads were assembled *de novo* using Shovill (v1.1.0), which integrates SPAdes for efficient genome assembly of Illumina data sets. Genome annotations were generated using both Prokka (v1.14.6) and Bakta (v1.9.4) to ensure comprehensive functional prediction.

Comparative genomic alignments were visualized using BRIG (v0.95), PROKSEE, and Easyfig (v.2.2.5) platforms. To identify genetic variations between P1-F-LS and P1-F-LR isolates, SNPs and insertion/deletion events (indels) were detected using Snippy (v4.6.0) (https://github.com/tseemann/snippy), and core genome SNPs were determined using Snippy-core. Functional annotation of SNP-associated genes was performed using the eggNOG v5 public database. Antimicrobial resistance genes were screened using ABRicate (v1.0.1) with ResFinder database and AMRFinderPlus (v3.12.8); both run on the Galaxy Europe platform with default settings. Mobile genetic elements were detected using ICEberg (v3.0), PlasmidFinder (v2.1.6), ISEScan (v1.7.3), and STR to bed (v.1.3.3) to identify integrative and conjugative elements (ICEs), plasmids, insertion sequences, and short tandem repeats, respectively. Integration sites of ICEs were analyzed using the complete genome of *Finegoldia* sp. strain WGS1513 (accession number GCF_043162015.1; chromosome size 1,883,469 bp) selected as a reference based on both availability of a fully assembled chromosome sequence and the presence within this sequence of all genomic regions flanking the three ICEs identified in our study.

### Genomic identification and strain relationship

Species assignment was assessed using whole genome-based methods, including average nucleotide identity, based on BLAST (ANIb) and *in silico* DNA–DNA hybridization (*is*DDH). ANIb values were calculated using the JSpecies Web Server (http://jspecies.ribohost.com/jspeciesws), and *is*DDH values were obtained *via* the Genome-to-Genome Distance Calculator (GGDC 3.1). Comparisons were made against the genomes of *Finegoldia magna*-type strain, DSM 20470^T^ (accession no. GCF_003182075.1), *Finegoldia dalianensis*-type strain, LY240594^T^ (accession no. GCF_046255015.1), and *S. epidermidis*-type strain, ATCC 14990^T^ (NZ_CP035288.1).

### Genetic support for linezolid resistance

LZD resistance-associated genes (*cfr*, *optrA*, and *poxtA*) were screened using ResFinder and AMRFinderPlus databases. Mutations in ribosomal protein genes *rplC* (encoding 50S ribosomal protein L3), *rplD* (L4), and *rplV* (L22), associated with LZD resistance were identified through SNP analysis with the *Finegoldia* sp. P1-F-LS genome taken as the reference or the *S. epidermidis* type strain, both LZD-susceptible. Mutations in domain V of the 23S rRNA gene were assessed by SNP analysis through sequence alignment using Integrative Genomics Viewer (IGV). For *Finegoldia* sp. strains, we referred to the complete 23S ribosomal RNA sequence (*rrnA* operon) of *F. magna* strain ATCC 29328 (accession no. AB109769.1) derived from the reference publication of Todo et al. (22), whereas *S. epidermidis* strain was compared to the complete 23S rRNA sequence from the *S. epidermidis*-type strain. Additionally, mutation positions were reported relative to the *rrlH* gene of the *Escherichia coli* strain, K-12 MG1655 (accession no. U00096.3) to enable standardized cross-species positional comparison.

## RESULTS

### Clinical and microbiological contexts of linezolid-resistant *Finegoldia* sp. isolates

The clinical and microbiological data for the three patients included in this study are presented in Data S1. In short, the three LZD-resistant *Finegoldia* sp. isolates under study were recovered from bone biopsies in three patients with complex clinical histories of polymicrobial infections, each having undergone surgical revision and received at least 4 weeks of LZD at standard dose of 600 mg twice daily (Data S1).

### Antimicrobial susceptibility profiles

Antibiotic susceptibility results are presented in Table 1. All *Finegoldia* sp. isolates were susceptible to β-lactams, rifampicin, metronidazole, and vancomycin but resistant to moxifloxacin. All exhibited an MLSb resistance phenotype, constitutive in the three clindamycin-resistant isolates, and inducible in the clindamycin-susceptible P1-F-LS strain. Among LZD-resistant isolates, two showed high-level resistance with MICs of LZD >256 mg/L, and the third displayed a lower MIC of 8 mg/L while being resistant to chloramphenicol suggestive of a PhLOPS_A_ phenotype (i.e., resistance to phenicols, lincosamides, oxazolidinones, pleuromutilins, and streptogramin A), although pleuromutilins were not tested. Although no breakpoints are currently available for interpretation, the MICs of tedizolid were consistently far lower than those of LZD (Table 1). The P3-SE-LR strain was methicillin-resistant, displayed a constitutive MLSb resistance phenotype, along with a KTG profile of aminoglycoside resistance, and was resistant to levofloxacin, fusidic acid, trimethoprim-sulfamethoxazole, and rifampicin. It exhibited high-level resistance to both LZD and tedizolid (MIC values >256 mg/L).

**TABLE 1:** Antimicrobial susceptibility profiles and resistome of the four *Finegoldia* sp. strains in the study^*a*^^,^^*b*^

	P1-F-LS	P1-F-LR	P2-F-LR	P3-F-LR
**Minimal inhibitory concentration (MIC, mg/L)**				
Amoxicillin	0.125	0.094	0.125	0.094
Amoxicillin-clavulanic acid	0.016	0.016	0.016	0.023
Piperacillin-tazobactam	0.032	0.032	0.064	0.047
Imipenem	0.032	0.023	0.032	0.032
Clindamycin	0.125	**>256**	**>256**	**>256**
Linezolid	1.5	**>256**	**>256**	**8**
Tedizolid^*c*^	0.5	1.5	1	0.38
Metronidazole	0.25	0.25	0.25	0.125
Rifampicin	0.25	0.125	0.19	0.25
Moxifloxacin	**12**	**8**	**3**	**6**
Vancomycin	0.25	0.25	0.19	0.125
**Inhibition zone diameter (mm)**				
Chloramphenicol	26	**6**	**6**	**18**
**Resistance gene (% id. [% cov.])**				
*cfr*(C)	–	97.25 (95.78)	97.25 (95.78)	97.25 (95.78)
*erm*(A)	100 (100)	100 (90.53)	99.59 (100)	99.59 (100)
*erm*(B)	–	–	–	100 (100)
*tet*(M)	–	–	100 (100)	–
**Literature-reported mutations linked to linezolid resistance**				
*rplD* mutation (L4)	ref.	G71D	G71D	–
23S rRNA domain V mutations	^*d*^	^*d*^	^*d*^	^*d*^

^*a*^ Bold values indicate resistance according to the 2025 CA-SFM guidelines (21).

^*b*^ The *cfr*(C) gene confers resistance to lincomycin, clindamycin, dalfopristin, pristinamycin IIA, linezolid, chloramphenicol, and florfenicol. The *erm*(A) and *erm*(B) genes confer resistance to lincomycin, clindamycin, erythromycin, quinupristin, and pristinamycin IA. The *tet*(M) gene confers resistance to tetracycline, doxycycline, and minocycline.

^*c*^ No breakpoints currently available to interpret the susceptibility of anaerobes to tedizolid.

^*d*^ See Table S1 for all detected 23S rRNA domain V mutations, none of which have been previously associated with linezolid resistance; ref.: strain used as reference for mutation detection; % id.: percentage of identity; % cov.: percentage of coverage; –:, not detected.

### Genomic relationship between *Finegoldia* sp. strains and identification

Genomic comparisons confirmed that the three patients were infected with unrelated *Finegoldia* sp. strains (>30,000 SNPs). By contrast, genomic comparisons revealed that strains P1-F-LS and P1-F-LR, both isolated 8 months apart from P1, were closely related, differing by two deletions, three insertions, and 32 core SNPs. Of these, 24 SNPs affected coding regions across 21 distinct genes, most of them being associated with replication, recombination and repair, or inorganic ion transport and metabolism (data not shown). Due to the limited genomic data available for the *Finegoldia* genus, a clear interpretation of the clonal relationship between strains remains challenging. However, the clinical history of Patient P1, combined with the high genomic similarity between the isolates, supports a clonal origin of the two strains and suggests *in vivo* emergence of LZD resistance.

All four *Finegoldia* sp. strains were initially identified as *F. magna* by MALDI-ToF MS. However, when their whole genome sequences (WGSs) were compared to those of the type strains of *F. magna* (GCF_003182075.1) and of the recently described species *F. dalianensis* (GCF_046255015.1), none exceeded the species threshold of 70% in *is*DDH, suggesting that all four isolates probably belong to undescribed *Finegoldia* species. Although ANIb clearly confirmed that the two isolates from P1 (P1-F-LS and P1-F-LR) do not belong to the *F. magna* species with values of 90.4 and 90.2%, respectively, interpretation was more challenging for the other two strains. Indeed, P2-F-LR and P3-F-LR exhibited ANIb values of 95.0 and 95.2%, respectively, when compared to the *F. magna* type strain, which fall within the borderline range for species delineation (commonly accepted 95–96% threshold) (Table 2). As *is*DDH remains the gold standard for species identification, none of the four isolates could be reliably classified as *F. magna* in this study. Altogether, these results suggest that all three patients were each infected with a distinct, previously undescribed *Finegoldia* species.

**TABLE 2:** Genome metrics (*is*DDH (up) and ANIb (down) values) between whole-genome sequences of the four *Finegoldia* sp. strains in the study and type strains of *Finegoldia magna* and *Finegoldia dalianensis^a^^,^^b^^,^^c^^,^^d^*

*is*DDH	*F. magna* ^T^	*F. dalianensis* ^T^	P1-F-LS	P1-F-LR	P2-F-LR	P3-F-LR
ANIb						
*F. magna* ^T^	100	68.5	42.8	43.2	66.2	68.3
*F. dalianensis* ^T^	**95.2**	100	42.1	42.7	64.4	64.5
P1-F-LS	90.4	90.3	100	**99.8**	42.9	43.1
P1-F-LR	90.2	90.3	**99.9**	100	44.1	42.8
P2-F-LR	**95**	94.8	90.6	90.7	100	62.1
P3-F-LR	**95.2**	94.7	90.5	90.1	94.3	100

^*a*^ *Finegoldia magna*-type strain DSM 20470^T^ (=ATCC 14904^T^) (accession number GCF_003182075.1); *Finegoldia dalianensis*-type strain LY240594^T^ (=GDMCC 1.4375^T^ = KCTC 25838^T^) (accession number GCF_046255015.1).

^*b*^ *is*DDH: *in silico* DNA-DNA hybridization; ANIb: average nucleotide identity based on BLAST; P1, P2 and P3: patients 1, 2, and 3, respectively; F: *Finegoldia* sp.; LS and LR: linezolid-susceptible and linezolid-resistant, respectively.

^*c*^ Bold type indicates values above the thresholds for species delineation of 70% for *is*DDH and 95% for ANIb.

^*d*^ The gray diagonal separates ANIb values (above the diagonal) from isDDH values (below the diagonal), thereby facilitating the readability of the table.

### Resistome of linezolid-resistant strains

The *erm*(A) gene encoding 23S rRNA (adenine(2058)-N (6))-methyltransferase was identified in all *Finegoldia* sp. strains, consistent with their MLSb phenotype, either constitutive or inducible (Table 1). In addition, strain P2-F-LR harbored a repUS43-type plasmid carrying the *tet*(M) gene, which encodes a ribosomal protection protein conferring tetracycline resistance, whereas strain P3-F-LR harbored the *erm*(B) gene (Table 1). All LZD-resistant *Finegoldia* sp. isolates possessed identical Cfr(C) protein sequences, which differed from the *Campylobacter coli* reference Cfr(C) protein (accession no. WP_111690898.1) with 95.78% coverage and 97.25% identity (Fig. S1). Notably, this gene was absent from the complete or draft *Finegoldia* genomes available in the National Center for Biotechnology Information’s database to date (22 October 2025).

In P1-F-LR and P2-F-LR, both of which exhibited high-level LZD resistance (MIC >256 mg/L), a G→A point mutation at position 212 in the *rplD* gene (encoding the 50S ribosomal protein L4) resulted in a G71D amino acid substitution. This mutation was absent in P3-F-LR (MIC = 8 mg/L) (Table 1). No mutations were identified in the *rplV* and *rplC* genes, which encode the 50S ribosomal proteins L22 and L3, respectively. An extended region of the 23S rRNA domain V, including flanking sequences (positions 2,000–2,900, *E. coli* numbering), was analyzed to assess its potential involvement in LZD resistance in *Finegoldia* spp. Eleven nucleotide variations were identified across *Finegoldia* isolates compared with *F. magna* ATCC 29328; none were present in all *rrn* operons, and none have previously been associated with LZD resistance. No mutation was shared between high-level LZD-resistant isolates, and P1-F-LR shared all variants with its paired susceptible isolate. Seven mutations were detected in the moderately resistant P3-F-LR isolate, three of which were shared with P2-F-LR (Table S1). Although no clear association between 23S rRNA domain V mutations and LZD resistance was observed, further functional studies, including targeted mutagenesis, are warranted to determine their potential role in LZD resistance.

No discrepancies were observed between genotypic resistance profiles and phenotypic AST results.

In *S. epidermidis* strain P3-SE-LR, the resistance genes accounted for the resistance phenotype described above, including *mecA* for methicillin resistance, *aac(6′)-Ie/aph(2″)-Ia* for aminoglycoside resistance, and *fusB* for fusidic acid resistance. Moreover, two non-synonymous mutations in the *rplC* gene (Q136L and M156T), a G69R substitution and two glycine residue insertions at positions 71 and 72 in the *rplD* gene and a G2576T mutation (*E. coli* numbering) present in all 23S rRNA gene copies were identified. All of these mutations have previously been associated with LZD resistance (13, 23, 24). No *cfr* gene was found in strain P3-SE-LR, excluding the possibility that it was the source of the *cfr*(C) gene detected in the co-isolated *Finegoldia* sp. P3-F-LR strain in Patient P3.

### Genetic environment of the *cfr*(C) gene

**Fig 1:**
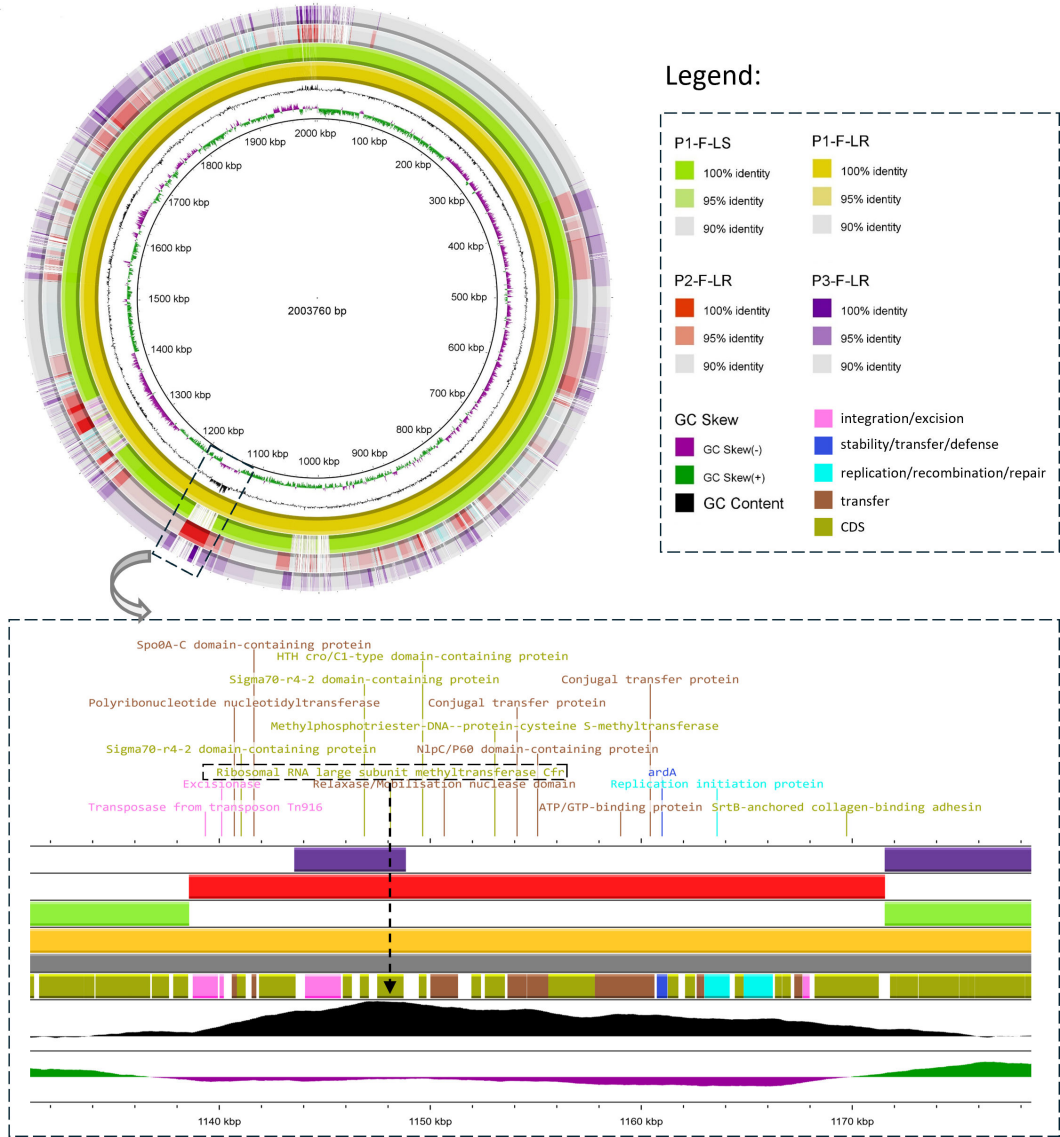
Comparative analysis of genome sequences using circular visualization showing the ICE present in LZD-resistant strains in patients P1 and P2. Comparative genome alignments were visualized using BLAST Ring Image Generator (BRIG, version 0.95). A circular diagram was generated with P1-F-LR (2,003,760 bp) as the reference genome. The innermost ring represents the GC content of the reference genome. The successive rings correspond to the genomes of P1-F-LR (reference), P1-F-LS, P2-F-LR, and P3-F-LR (outermost). The ICE (highly homologous to elements found in *Clostridioides difficile*) is shared by strains P1-F-LR and P2-F-LR but absent in P3-F-LR. Genomic positions are labeled in kilobase pairs (kbp). Sequence identity is color-coded: dark shades represent 100% identity, lighter shades 95%, and light gray 90%. Regions of high similarity appear darker, while gaps or lighter areas reflect genomic divergence or absence. Dashed boxes highlight the regions containing *cfr*(C)-carrying ICEs. Contiguous genes are represented by a color according to their function.

**Fig 2:**
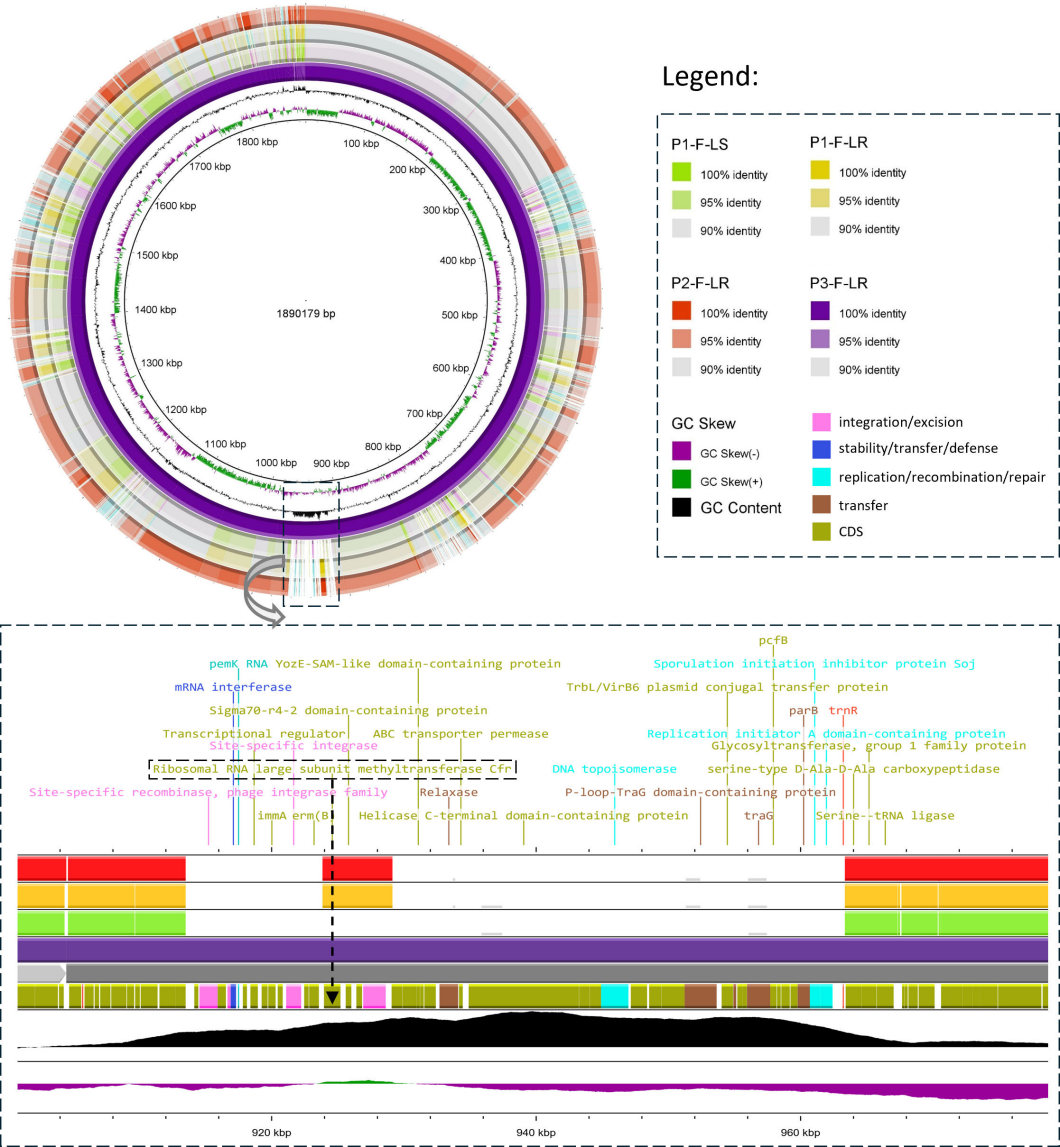
Comparative analysis of genome sequences using circular visualization showing the ICE present in LZD-resistant strain in Patient P3. Comparative genome alignments were visualized using BLAST Ring Image Generator (BRIG, version 0.95). A circular diagram was generated with P3-F-LR (1,890,179 bp) as the reference. The innermost ring represents the GC content of the reference genome. The rings display P3-F-LR (reference), followed by P1-F-LS, P1-F-LR, and P2-F-LR (outermost). This figure reveals that P3-F-LR carries a distinct ICE (highly similar to one found in *Faecalibacterium taiwanense*), underscoring differences in the genetic background of LZD resistance among these *Finegoldia* isolates. Genomic positions are labeled in kilobase pairs (kbp). Sequence identity is color-coded: dark shades represent 100% identity, lighter shades 95%, and light gray 90%. Regions of high similarity appear darker, whereas gaps or lighter areas reflect genomic divergence or absence. Dashed boxes highlight the regions containing *cfr*(C)-carrying ICEs. Contiguous genes are represented by a color according to their function.

**Fig 3:**
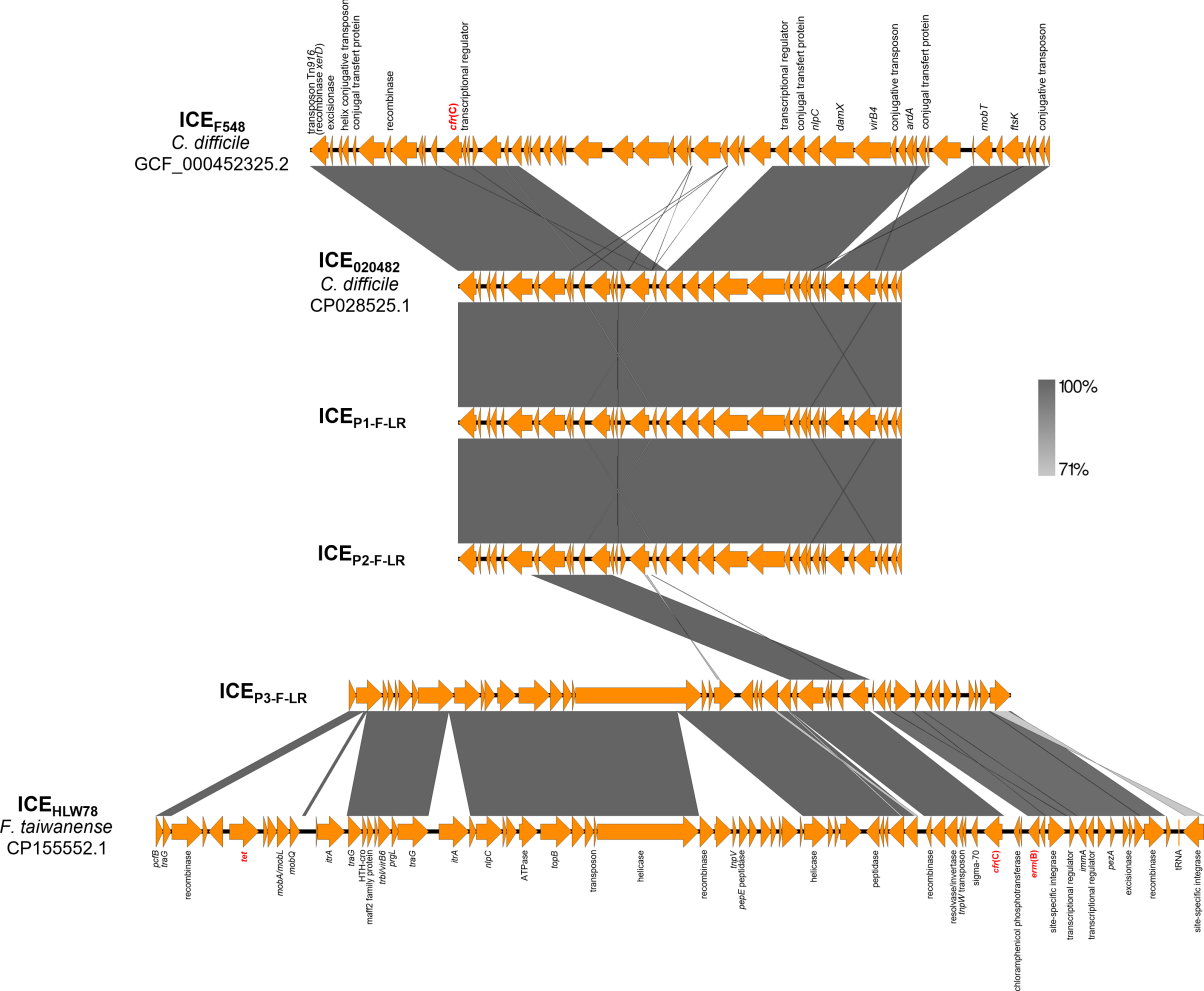
Genetic environments of the *cfr*(C) gene in LZD-resistant strains P1-F-LR, P2-F-LR, and P3-F-LR, in comparison with the ICE_F548_-type organization (29, 30). Orange arrows depict coding sequences. Antibiotic resistance genes are indicated in red. Dark gray shading indicates regions with 100% nucleotide sequence identity. The names of the strains represented are listed on the left. P1-F-LR and P2-F-LR share highly similar ICE structures (F548-like ICE), whereas P3-F-LR harbors a distinct ICE.

Although the same *cfr*(C) gene was present in all LZD-resistant *Finegoldia* sp. isolates, its genetic environment varied among strains (Fig. 1). In strain P1-F-LR, a 29,237-bp type IV secretion system (T4SS)-type ICE was identified (ICE_P1-F-LR_) carrying the *cfr*(C) gene. This ICE showed 100% coverage and 99.99% identity with several *Clostridioides difficile* complete genomes (strains 020688, 020482, 020711 (25), CDI-01 (26), DSM 104450 (27), and DSM 29688 (28)) and 100% coverage with 99.97% identity to the *Clostridium perfringens* complete genome of strain 19TSBNCP (29) (Fig. 1). A similar ICE was detected in P2-F-LR (ICE_P2-F-LR_), differing from ICE_P1-F-LR_ by only two SNPs. In contrast, strain P3-F-LR harbored a distinct 43,642 bp T4SS-type ICE (ICE_P3-F-LR_), showing 98.86% coverage and 99.5% identity with the *Faecalibacterium taiwanense* complete genome of strain HLW78 (accession no. CP155552.1) (Fig. 2). Both ICE_P1-F-LR_ and ICE_P2-F-LR_ shared highly conserved structure, with the ICE detected in *C. difficile* strain 020482 (accession no. CP028525.1) and with regions of ICE_F548_ (48,752 bp) described in *C. difficile* strain F548 (30) (Fig. 3). These ICEs, therefore, exhibited an F548-like structural organization. Comparative structural features between the ICEs identified in this study and those previously described by Candela et al. and Zhang et al. are shown in Fig. 3 (29, 30). Analysis using the complete genome of *Finegoldia* sp. strain WGS1513 as a reference revealed that the three ICEs integrate at distinct chromosomal locations, demonstrating the absence of a common integration hotspot (Fig. S2). ICE_P1-F-LR_ was inserted at position 286,646 bp, between genes encoding a hypothetical protein and an ATPase; ICE_P2-F-LR_ was located at position 1,272,711 bp within the *galectin* gene; and ICE_P3-F-LR_ was inserted at position 1,432,262 bp within a tRNA-Arg gene (*trnR*). No insertion sequences were detected in the genomic regions flanking the ICEs, supporting the hypothesis that ICE integration was not plasmid-mediated (31). No repeated elements were detected flanking ICE_P1-F-LR_ and ICE_P2-F-LR_, both of which begin with a Tn*916* transposon (tyrosine recombinase XerD) known for its low integration specificity and lack of flanking direct repeats (32). In contrast, ICE_P3-F-LR_ was inserted within a repeated motif of the tRNA-Arg gene (5′-GGGGGG-3′) (Fig. S2). ICEs identified in this study were integrated into the host genome and encoded a conserved conjugation machinery, including a T4SS homologous to that found in conjugative plasmids, which mediates DNA transfer between bacterial cells. The presence of recombinase genes suggested a potential for excision and horizontal transfer.

## DISCUSSION

LZD resistance remains rare overall and, among aerobes, has been documented in *Staphylococcus aureus*, coagulase-negative staphylococci, *viridans* group streptococci, *Enterococcus faecium*, and *Enterococcus faecalis* (13, 14). In anaerobes, acquired LZD resistance has mostly been reported in *Bacteroides* spp. (17, 33, 34) and *Clostridioides difficile* (35–38) and sporadically in other Gram-negative and -positive anaerobic species (35, 36, 39, 40). In these species, LZD resistance-encoding genes were mostly *cfr*-like genes found on plasmids or on the chromosome with insertion elements in strains that might also harbor mutations in protein L3 and/or L4-encoding genes (29, 37, 38, 41–44). In GPAC, LZD resistance remains exceedingly rare (3, 4, 35, 36). No strains with high-level resistance to LZD have been previously reported, and none of the previous studies have characterized the genetic support for LZD resistance in GPAC. In this context, we provided a comprehensive clinical, microbiological, and genomic characterization of LZD-resistant *Finegoldia* sp. strains isolated from three unrelated cases of bone infection, all treated by LZD, and otherwise presenting lower MICs of tedizolid, another oxazolidinone with broad activity against anaerobes (39). The resistant strains were isolated after 1 to 7 months of LZD therapy, and, in two patients, following prior isolation of LZD-susceptible *Finegoldia* sp. strains. In one patient, WGS analysis supported the hypothesis of *in vivo* emergence of LZD resistance, consistent with previous studies, including our previous work on *S. aureus* chronic colonization in patients with cystic fibrosis. In that work, the literature review highlighted highly variable LZD selective pressure times before resistance emerged depending on the study under consideration (13 to 453 days of treatment; mean time of 128 days) (45). At the same time, our findings underlined that MALDI-ToF MS, although widely regarded as an excellent tool for GPAC identification (46), was limited in accurately identifying *F. magna* due to the uncharacterized species diversity within the genus. This highlights the need for a formal description of novel *Finegoldia* species and their inclusion in mass spectrometry databases. The three LZD-resistant *Finegoldia* strains harbored the same *cfr*(C) gene, a LZD-resistance-associated gene previously observed in two *Bacteroides fragilis* isolates from chicken (LZD MIC = 8 mg/L) (41), a *Clostridium bolteae* isolate from a pelvic abscess (LZD MIC = 16 mg/L) (30), a *C. perfringens* strain from the intestinal contents of a cow (LZD MIC = 16 mg/L) (29), and described as the *cfr* gene, the most commonly detected gene among 2,134 *C. difficile* genomes (4%) (47). However, phylogeny-based studies suggest that transmissible oxazolidinone resistance determinants are present in a much wider variety of microorganisms than the repertoire of species identifiable from published studies (30, 47, 48). Notably, *Finegoldia* spp. were absent from these studies, including the largest study by Kardos et al. where GPAC were represented by *Ruminococcus* sp. carrying either *cfr*(C) or *cfr*(B) only (48). In this study, the *Finegoldia* sp. strains displayed distinct levels of resistance related to distinct genetic mechanisms. High-level resistance in P1-F-LR and P2-F-LR was associated with both ribosomal target methylation and point mutations, whereas P3-F-LR, with a lower level of resistance, displayed a unique resistance mechanism mediated by the *cfr*(C) gene. The combination of LZD resistance mechanisms is an extremely rare observation in anaerobes but, as observed in aerobes, can result in higher resistance levels (15).

In anaerobes, the *cfr*(C) gene has previously been described on diverse mobile genetic elements, either on plasmid (pCd13-Lar), transposons (Tn*6994*, Tn*6314*) or on various types of ICEs (i.e., ICE_DA275_, ICE_F548_, F548-like ICE, ICE_90B3_ and ICEC*d-cfr*(C)) (15, 30, 41, 42). Here, we showed that the genomic environments of the *cfr*(C) gene differed depending on the strain, with the *cfr*(C) gene being carried by two distinct ICEs in the three LZD-resistant strains. One of them, F548-like ICE, had been previously described in *Clostridium* spp. (47), whereas the second one (not yet described in the literature) was found to have a sequence homologous with one found in *Faecalibacterium taiwanense* and was associated with the *cfr*(C) gene and LZD resistance for the first time. Although conjugation experiments have not been performed in this study, the distinct ICE gene structures, their description in unrelated taxa, and the belonging of the three *cfr*(C)-harboring strains to three distinct lineages in the genus *Finegoldia* suggested the transferability of the ICEs and likely reflected three independent events of *cfr*-carrying ICE acquisition. This was also supported by the identification of three distinct chromosomal integration sites when mapped onto the WGS1513 reference genome. We were, however, unable to identify the source for the mobilizable *cfr*(C) gene as the only LZD-resistant strain co-isolated from these patients had independent resistance mechanisms. However, mobile LZD-resistance genes like *cfr* genes have been identified from highly diverse sources, including animal and environmental ones like manure (49), suggesting that the patients might have had endogenous, as well as exogenous, contamination through one of these other reservoirs of antibiotic-resistant bacteria and antibiotic-resistant genes.

In conclusion, the emergence of LZD resistance described in this study in three patients treated with LZD for bone infections involving *Finegoldia* sp. confirms the importance of performing AST when managing infections involving this genus, as emphasized by Walser et al. (50). AST should also be repeated during follow-up to track for any emergence of resistance under treatment. When LZD resistance is detected, tedizolid may represent a potential therapeutic alternative. Recent findings have also shown that LZD-resistant *Enterococcus* strains can display enhanced bacterial growth and fitness under antibiotic selective pressure, an observation that warrants further investigations in anaerobes (51).

## Data Availability

Raw sequencing data and genome assemblies have been deposited in GenBank under the BioProject accession number PRJNA1331717 (BioSample accession numbers SAMN51640392, SAMN51640393, SAMN51640394, SAMN51640395, and SAMN51640396 for P1-F-LS, P1-F-LR, P2-F-LR, P3-F-LR, and P3-SE-LR strains, respectively).

## SUPPLEMENTAL MATERIAL

Supplemental material aac.01702-25-s0001.docx Data S1; Fig. S1 and S2; Table S1.

ASM does not own the copyrights to Supplemental Material that may be linked to, or accessed through, an article. The authors have granted ASM a non-exclusive, world-wide license to publish the Supplemental Material files. Please contact the corresponding author directly for reuse.
