# Supplementary material for: Molecular basis of linezolid resistance in Finegoldia spp. from orthopedic infections
Source: Antimicrob Agents Chemother. 2026 Feb 26;70(4):e01702-25. doi: 10.1128/aac.01702-25 (PMC13041314; doi:10.1128/aac.01702-25)
Supplement: Supplemental material — Data S1; Fig. S1 and S2; Table S1. [file aac.01702-25-s0001.docx]

**SUPPLEMENTARY DATA**

**Supplementary data 1.** Clinical and microbiological context of linezolid-resistant *Finegoldia* sp. isolates.

The clinical histories of the three patients were complex. To preserve clarity and anonymity, only the clinical and microbiological data strictly relevant to this study, are presented, excluding demographic details, timelines, and exhaustive clinical data. A summary of the key information (general clinical context, microbiological history: prior isolation of linezolid (LZD)-susceptible *Finegoldia* sp. strain, co-isolated species and their LZD susceptibility when tested, and antibiotic selective pressure) is presented below, with strains further studied highlighted in bold type.

Patient P1 had a long-standing chronic polymicrobial bone infection following an ankle fracture requiring ankle arthrodesis and complicated by persistent pain, successive surgeries, and several antimicrobial regimens including daptomycin, cefotaxime, ofloxacin, clindamycin and LZD. The LZD-resistant Finegoldia sp. strain (**P1-F-LR**), identified as Finegoldia magna by matrix-assisted laser desorption/ionization time-of-flight mass spectrometry (MALDI-Tof MS), was isolated after two LZD treatment courses of three and four months, respectively. Two prior Finegoldia sp. isolates (one isolated one month before any LZD exposure and one isolated between the two LZD regimens) were susceptible to LZD. Only the earliest isolate (**P1-F-LS**), recovered eight months before the LZD-resistant P1-F-LR, was available for comparative genomic analysis. Among the other bacterial species isolated from successive bone samples (Staphylococcus aureus, Staphylococcus caprae, Staphylococcus epidermidis, Staphylococcus capitis, Enterococcus faecalis, and Enterococcus gallinarum), none of the strains subjected to antimicrobial susceptibility testing (AST) displayed LZD resistance except for Peptoniphilius asaccharolyticus co-isolated with P1-F-LR, which was not available for further analysis.

Patient P2 was a diabetic patient presenting with a necrotizing diabetic foot polymicrobial infection involving *S. aureus* and anaerobes (*F. magna*, *P. asaccharolyticus* and *Actinomyces turicensis*) requiring toe amputation. Persistent necrosis despite clindamycin-ofloxacin therapy led to a second operation. Cultures from the bone biopsies showed a polymicrobial infection of E. faecalis, Bacteroides fragilis, Corynebacterium striatum and Finegoldia sp., identified as F. magna by MALDI-ToF MS, all susceptible to LZD. A six-week LZD regimen was initiated. Three months later, a bone biopsy sample still revealed a polymicrobial infection, including three Enterobacterales species and one LZD-resistant Finegoldia sp. strain (**P2-F-LR**).

Patient P3, also diabetic, underwent toe amputation complicated by a plantar abscess requiring repeat surgery and initial treatment combining ofloxacin and amoxicillin-clavulanic acid, based on the isolation of *Proteus mirabilis* and *Peptoniphilus harei* from operative samples. Due to interference with swarming of *P. mirabilis* on the culture agar medium incubated in anaerobiosis, AST could not be performed for *P. harei*. A four-week course of ofloxacin and LZD was prescribed. The following month, bone samples yielded a LZD-resistant *Finegoldia* sp. strain (**P3-F-LR**) and a LZD-resistant *S. epidermidis* strain (**P3-SE-LR**), confirming the diagnosis of infectious osteomyelitis.

**Supplementary figure 1.** Multiple sequence alignment of Cfr(C) proteins.

The protein sequences of the *cfr*(C) genes carried by integrative and conjugative elements (ICEs) from P1-F-LR, P2-F-LR, and P3-F-LR were aligned with *cfr*(C) sequences from *Finegoldia taiwanense* HLW78, *Clostridioides difficile* F548, *C. difficile* 020482, and *Campylobacter coli* (WP_111690898.1). The Cfr(C) proteins from P1-F-LR, P2-F-LR, P3-F-LR, *F. taiwanense* HLW78, and *C. difficile* F548 were strictly identical, each consisting of 416 amino acids. These sequences differed from the Cfr(C) protein of *C. difficile* 020482 by a single amino acid substitution at position 96 (W→C). In contrast, the Cfr(C) protein from *C. coli* was shorter (379 amino acids) and shared 95.78% sequence coverage with the 416-amino-acid Cfr(C) proteins, with 97.25% sequence identity to all sequences except that of *C. difficile* 020482, for which identity was 96.97%.

Amino acids that are identical across all protein sequences are shown in gray. In positions where at least one amino acid differs, the most conserved residues are shown in black, whereas divergent residues are highlighted in red. Red boxes indicate regions containing at least one amino acid substitution, while black dashed boxes delineate regions not covered by all protein sequences.

**Supplementary figure 2.** Chromosomal integration site of ICE_P1-F-LR_ (**A**), ICE_P2-F-LR_ (**B**) and ICE_P3-F-LR_ (**C**) in *Finegoldia* sp. strain WGS1513.


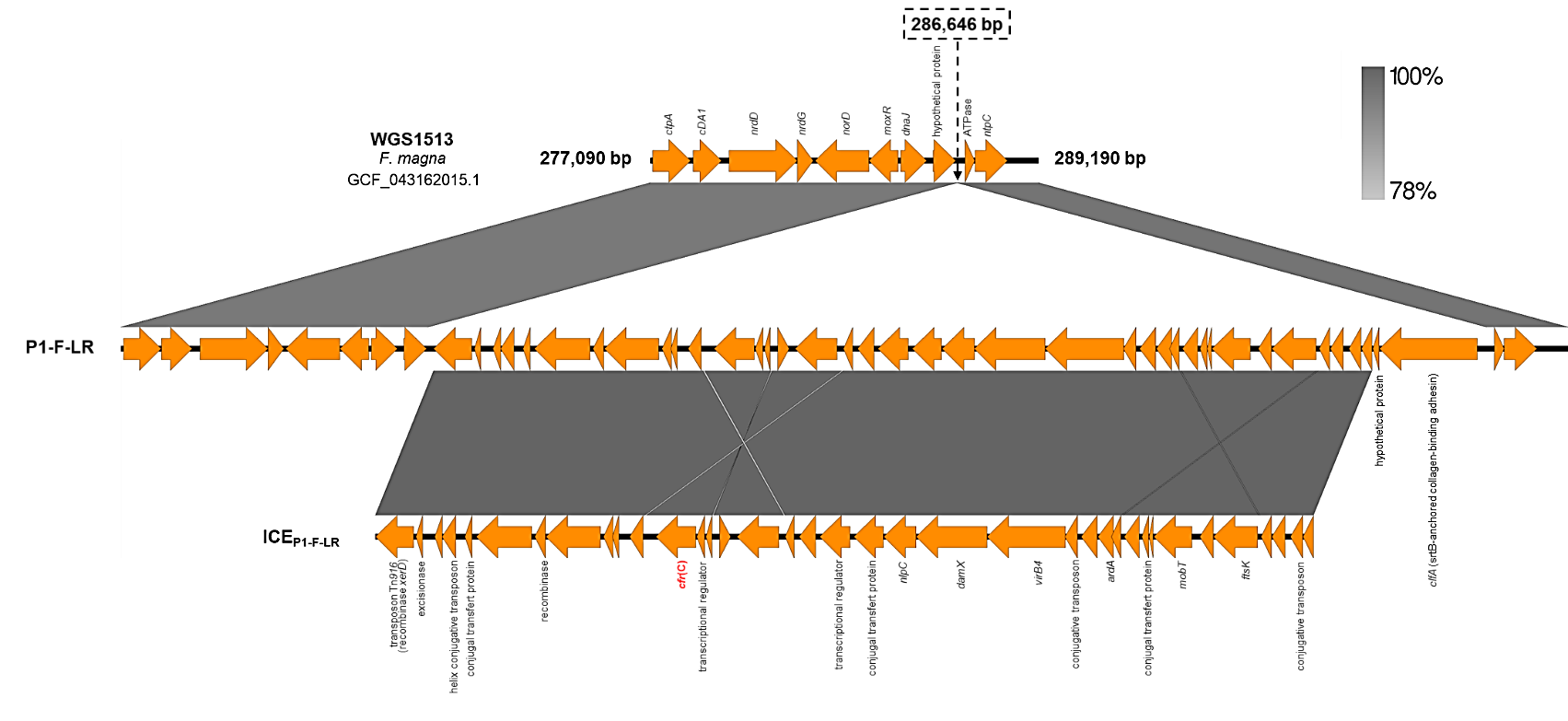

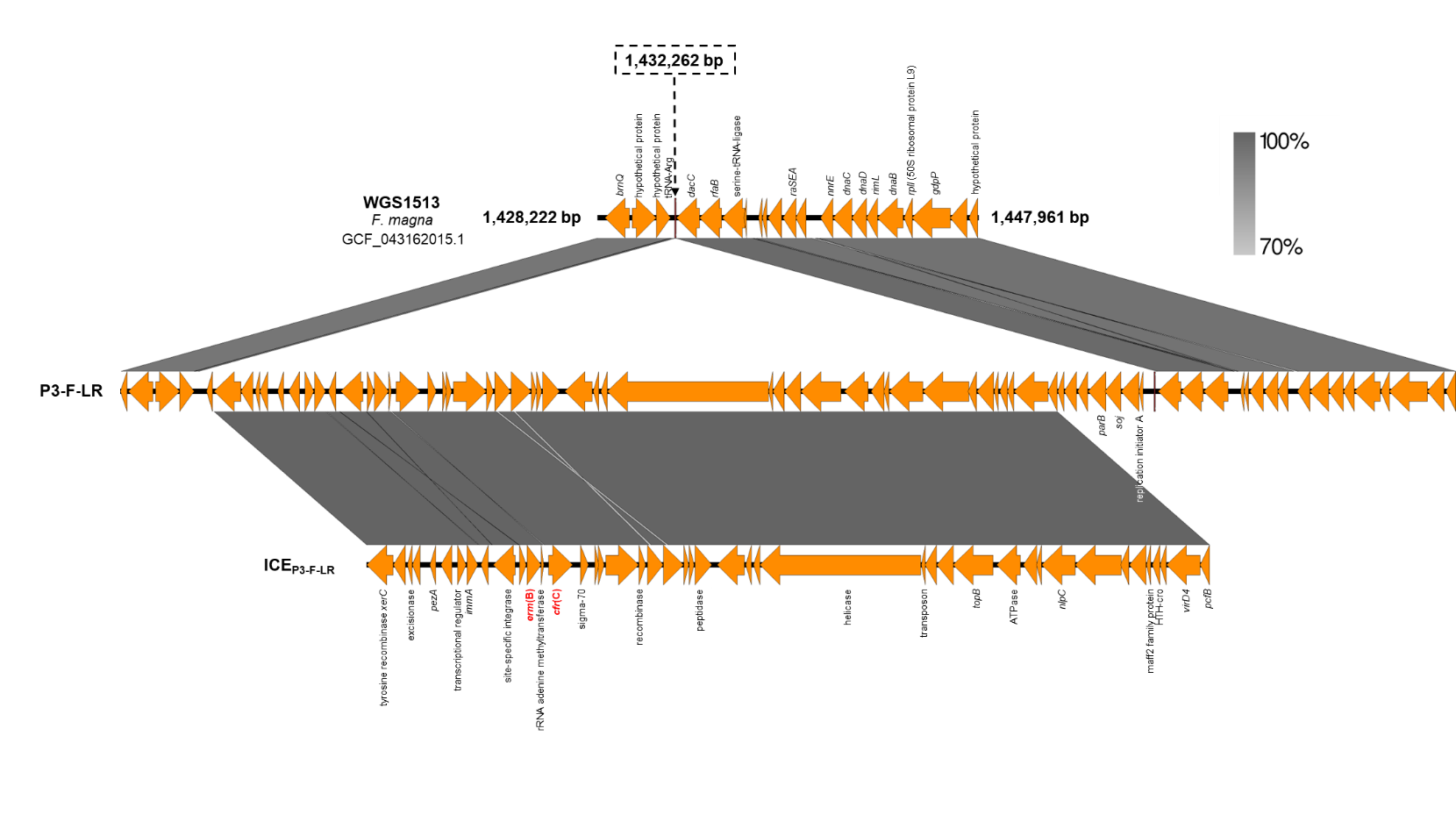

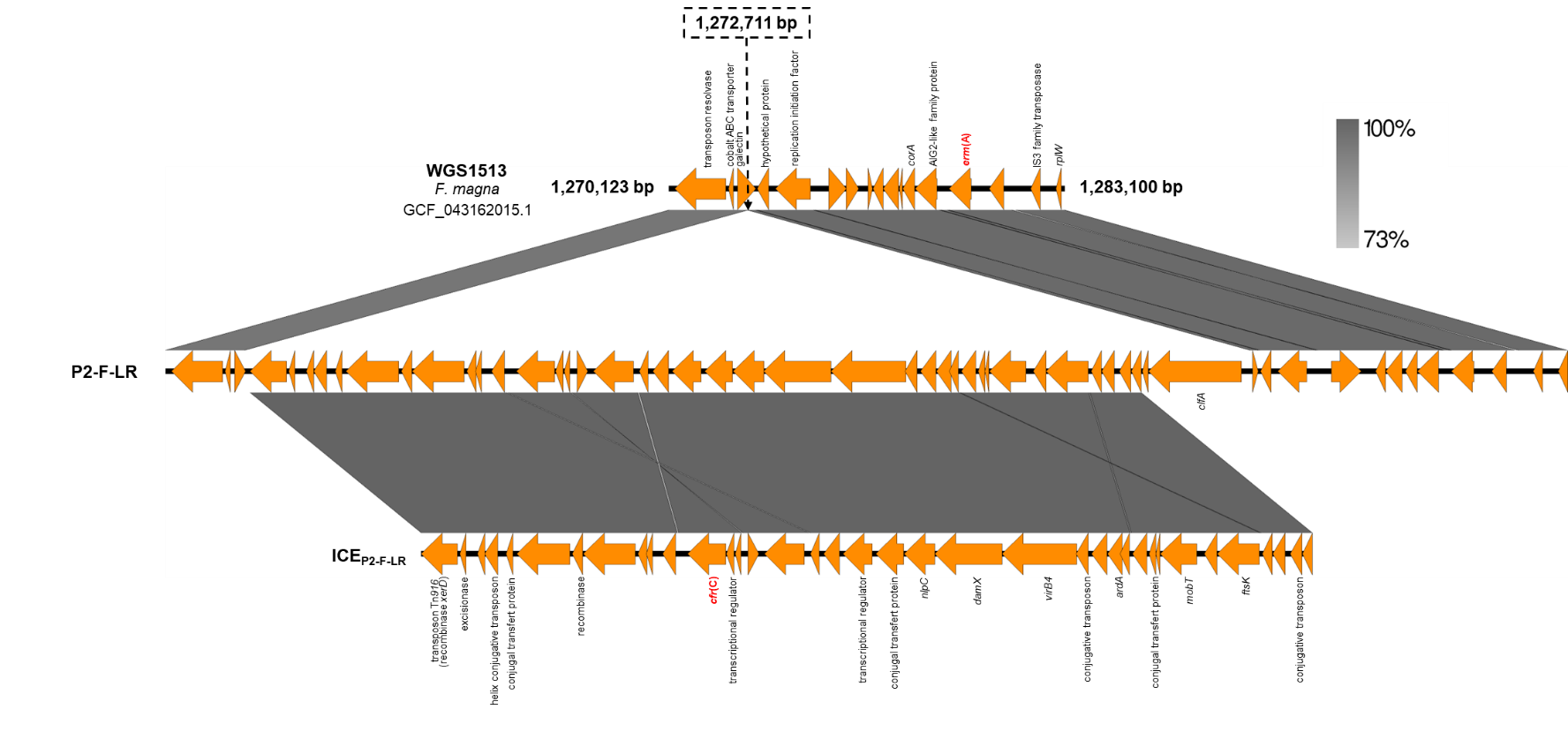


**A**

**B**

**C**

Orange arrows depict coding sequences, and antibiotic resistance genes are indicated in red. Dark gray shading indicates regions with 100% nucleotide sequence identity. The names of the strains represented are listed on the left. The ICE integration sites within the reference genome of *Finegoldia* sp. strain WGS1513 are indicated by dashed arrows. ICE_P1-F-LR_ was inserted at position 286,646 bp, between genes encoding a hypothetical protein and an ATPase (A); ICE_P2-F-LR_ was located at position 1,272,711 bp within the *galectin* gene (B); and ICE_P3-F-LR_ was inserted at position 1,432,262 bp within a tRNA-Arg gene (*trnR*) (C).

**Supplementary table 1.** Mutations in the 23S rRNA domain V of *Finegoldia* isolates compared to *F. magna* ATCC 29328.

| ***rrlH*  (*E. coli* K-12 MG1655)** | ***rrnA* (*F. magna*  ATCC 29328)** | **Reference** | **P1-F-LS** | **P1-F-LR** | **P2-F-LR** | **P3-F-LR** |
| --- | --- | --- | --- | --- | --- | --- |
| 2130 | 2247 | **G** |  |  | **T: 3/4 A: 1/4** | **G: 3/4  T: 1/4** |
| 2131 | 2248 | **A** |  |  |  | **A: 3/4  G: 1/4** |
| 2145 | 2262 | **C** |  |  |  | **C: 3/4 T: 1/4** |
| 2205 | 2323 | **A** |  |  | **A: 3/4  G: 1/4** | **G: 3/4  A: 1/4** |
| 2213 | 2331 | **G** |  |  | **A: 2/4 G: 2/4** | **A: 3/4  G: 1/4** |
| 2357 | 2475 | **T** |  |  |  | **A: 3/4  T: 1/4** |
| 2405 | 2523 | **G** | **G: 2/4 T: 2/4** | **G: 2/4 T: 2/4** |  |  |
| 2728 | 2846 | **T** | **T: 3/4 C: 1/4** | **T: 3/4 C: 1/4** |  |  |
| 2733 | 2851 | **G** |  |  | **G: 3/4 A: 1/4** |  |
| 2775 | 2893 | **G** |  |  |  | **G: 2/4 A: 2/4** |
| 2854 | 2954 | **A** | **A: 2/4  G: 2/4** | **A: 2/4  G: 2/4** |  |  |

Eleven SNP positions were identified across sequences of the *Finegoldia* isolates relative to the reference strain *F. magna* ATCC 29328. The LZD-resistant P1-F-LR and its susceptible counterpart P1-F-LS shared three variations (positions 2405, 2728, and 2854; *E. coli* numbering), with no mutation unique to P1-F-LR that could explain its high-level resistance. P2-F-LR carried four variations (positions 2130, 2205, 2213, and 2733) absent in P1-F-LR, suggesting that high-level resistance observed for these two strains was not driven by a common 23S rRNA gene alteration.

LZD-resistant strain P3-F-LR, which displayed lower LZD minimum inhibitory concentration than P1-F-LR and P2-F-LR, harbored seven variations (positions 2130, 2131, 2145, 2205, 2213, 2357, and 2775), three of which (2130, 2205, and 2213) were shared with P2-F-LR. Importantly, mutations were detected only in a subset of the four 23S rRNA gene copies in any isolate (1/4, 2/4, or 3/4), never in all copies simultaneously.
